# The 1968 Influenza Pandemic and COVID-19 Outcomes

**DOI:** 10.1101/2021.10.23.21265403

**Published:** 2021-10-25

**Authors:** Charles A Taylor, Christopher Boulos, Matthew J. Memoli

**Affiliations:** a School of International and Public Affairs, Columbia University. Corresponding author: 420 W 118th, New York, NY 10027; b Laboratory of Infectious Diseases, National Institute of Allergy and Infectious Diseases, National Institutes of Health

**Keywords:** COVID-19, 1968 Influenza Pandemic, Immunity, Persistence

## Abstract

Past pandemic experience at an individual or population level may affect health outcomes in future pandemics. In this study, we focus on how the influenza pandemic of 1968 (H3N2), which killed up to 100,000 people in the US, may have produced differential COVID-19 (SARS-CoV-2) outcomes. Our analysis finds that areas with high influenza-related mortality in 1968 experienced 1–2% lower COVID-19 death rates. We employ an identification strategy that isolates variation in COVID-19 rates across age cohorts born before and after 1968. Locales in the US with high 1968 influenza mortality have lower COVID-19 death rates among older cohorts *relative* to younger ones. The relationship holds using county-level and patient-level data, as well as data from hospitals and nursing homes. Results do not appear to be driven by systemic or policy-related factors that would affect a population, but instead suggest a potential individual-level response to prior influenza pandemic exposure. The findings merit substantial further investigation into potential biological, immunological, or other mechanisms that can account for these differential outcomes.

## Summary

**Background:**

Past pandemic experience at an individual or population level could affect health outcomes in future pandemics. In this paper we test whether people who lived through local outbreaks of the influenza pandemic of 1968 (H3N2), which resulted in 100,000 deaths in the US, experience better outcomes when infected by the SARS-CoV-2 virus.

**Methods:**

We employ econometric techniques on county-level and patient-level data, further validated by aggregated data from hospitals and nursing home, to explain differential COVID-19 outcomes. Given the cross-sectional nature of this study, we address endogeneity and omitted variable bias concerns in the relationship between the 1968 flu and COVID-19 outcomes by utilizing as our outcome variable the *difference* by age group in death rates, number of medical procedures, and hospital admissions. We choose the age cutoff based on whether someone was born before or after 1968 in an attempt to isolate those people more likely exposed to the 1968 pandemic.

**Findings:**

We find a persistent negative relationship between US locales’ exposure to the 1968 flu pandemic and COVID-19 mortality. This relationship is robust to various controls and holds across populations (i.e., county level aggregates, hospital admittees, and nursing home residents, subset of patients), as well as specifications that exploit age-based variation in exposure. Further analysis supports a possible individual-level mechanism—rather than policy-related, systemic, or population-level factors—as drivers of this phenomenon.

**Interpretation:**

Results suggest a potential biological or immunological mechanism that may mitigate the severity of COVID-19 in individuals who survived the 1968 flu pandemic. Further research should explore possible explanations for this phenomenon in the hopes of uncovering new avenues of prevention and treatment.

## 1. Introduction

The COVID-19 pandemic resulted in the global implementation of diverse public health and economic measures including social distancing and industrial and supply chain innovations, along with widespread emergency use of novel pharmaceuticals and vaccines. To evaluate the impact of these pandemic responses and to better understand how to prepare for and prevent future pandemics, there is now a broad effort to investigate the novel epidemiological and pathological nature of the SARS-CoV-2 virus in the hope of fully assessing the global impact of the disease—and, in particular, investigating the susceptibility of populations to illness.

One strand of research employs empirical analysis to explain the variability observed in COVID-19 outcomes among different populations globally. Attempting to catalogue and learn from epidemiological observation of the virus, previous work has presented evidence linking demographic, racial, contextual, social, and policy factors to the spread and severity of COVID-19 (1, 2, 3, 4, 5, 6, 7, 8, 9, 10, 11). In addition, since the beginning of the pandemic, there have been unequal responses globally and within the US with regard to mitigation behavior and compliance, even in the face of similar governmental policies and regulations. It has been proposed that these differing behaviors contribute to differential COVID-19 outcomes, but this has been difficult to demonstrate clearly.

Meanwhile, a substantial body of literature has explored genetic, immunological, and other biological factors linked to the severity and transmissibility of COVID-19 (12, 13, 14, 15). Research of this type includes genomic association studies, an investigation of sex differences in immunity, and a proposal that a major genetic risk factor can be traced to Neanderthal DNA (16, 17, 18). This category of work explores whether risk factors, biomarkers, and cross-reactivities can be identified and used to develop best practices in prophylaxis, testing, and management.

A third research thread explores how past public crises and their effects can persist, altering human and institutional responses in such a way that mediates the spread, morbidity, and mortality of COVID-19. Such work links COVID-19 outcomes with past wars or local experience of past pandemic response (19, 20). Rather than making claims about some particular factor or mechanism contributing to illness, these studies offer useful context that helps expose previously unexplored mechanisms. For instance, Lokshin et al. found that higher WWII mortality is associated with lower COVID-19 death rates, positing a link between institutional investment, social capital and pandemic response. Other studies have linked SARS exposure to lower COVID-19 mortality, which some attributed to mask wearing norms that made compliance with public health measures ‘easier’ (21, 22, 23).

This type of research has been applied to previous pandemics. One representative study links an HIV-resistant gene to exposure to the plague and smallpox (24, 25). Another study suggested that the 1918 influenza pandemic hastened the decline of tuberculosis (26). Others have linked excess youth mortality during the 1918 influenza pandemic to exposure to the 1889 ‘Russian Flu’ virus (27, 28).

### 1.1. The 1968 influenza pandemic

While many have looked to the 1918 influenza pandemic for insight into the ongoing epidemiological and economic effects of the COVID-19 pandemic(29, 30), there is a far more recent comparable public health crisis that had broad public impact. In September 1968, the US was confronted with a novel H3N2 influenza virus that originated in China and was dubbed the ‘Hong Kong Flu’ or ‘Mao Flu’.^1^ The death toll of the ensuing pandemic was comparable to that of the COVID-19 pandemic given the smaller US population of the time of around 200 million people, with 100,000 deaths in the US (compared to 400,000 COVID-19 deaths out of 330 million people as of January 2021). Globally, the 1968 flu pandemic resulted in between 1 to 4 million deaths. Paralleling the present day, the flu reached the White House, with both President Lyndon B. Johnson and Vice President Hubert Humphrey falling ill (31).

**Figure 1:**
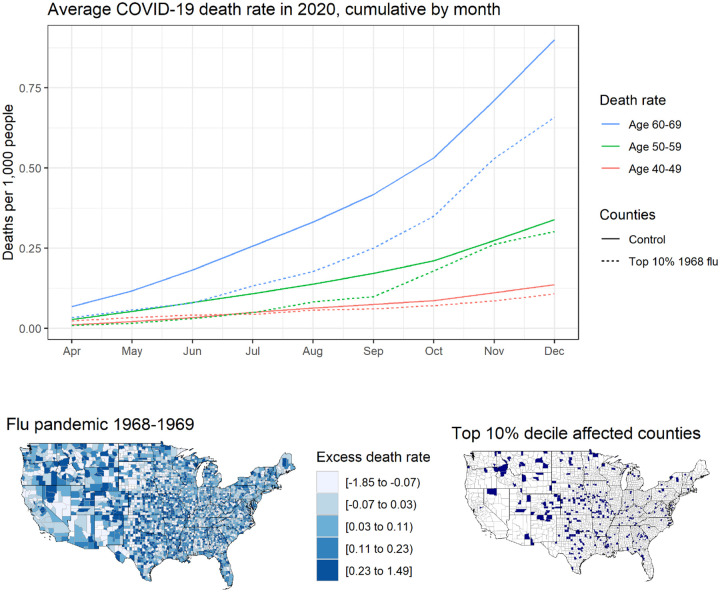
Chart shows average county-level cumulative COVID-19 death rates by age group and month for control counties, which include all counties except those in the top decile 10% of excess flu deaths in 1968, and those in the top 10%. Map shows distribution of influenza deaths per thousand at the county level estimated from CDC data of excess respiratory deaths in 1968 and 1969 relative to a baseline of 1970 and 1971.

Approximately one third of the US population was alive during the 1968 pandemic (32). As shown in the map in Figure 1, the 1968 flu spread nationwide. Some communities were hit especially hard; a contemporaneous report estimated that over 40% of the population of Milwaukee was infected (33). Several cities reported stress on local hospital systems attempting to manage the influx of patients (33, 34). Unlike SARS-CoV-2 today, the 1968 virus killed many young people, with approximately 40% of the flu-related deaths estimated to have been among those under 65 (35, 36).

Despite the widespread disruptions caused by the 1968 flu pandemic, the social, economic, and public health response at the federal level was somewhat muted, with much of the country operating as usual. Although 23 states underwent school and university closures, the US did not implement any broad social distancing or containment measures. Vaccines were eventually developed but not in time to blunt the initial spread of the virus (31, 37, 34).

The recency and scale of the 1968 pandemic make it a compelling event to employ historical analysis of the type seen in the third strand of literature cited above. Studying any enduring impact of this pandemic helps to further our understanding of the COVID-19 pandemic and how future pandemics can be mitigated. More immediately, it may motivate clinical research into immunological or biological responses to SARS-CoV-2 related to exposure to the 1968 pandemic.

## 2. Methods

### 2.1. Hypothesis and approach

We first test the hypothesis that a residual link exists between 1968 flu severity and current COVID-19 outcomes. We then assess potential factors that could explain such a relationship. While identifying precise mechanisms is outside the scope of this study, we perform several empirical tests to untangle potential *policy*, *social*, and *individual* channels. We limit the analysis to the end of 2020 to avoid potential confounding effects of differential vaccine uptake.

A *policy* channel would include any mechanism whereby an institution, in response to the 1968 flu pandemic, has put in place some deliberate action or policy that would have, regardless of its intention, influenced COVID-19 outcomes. Examples could be improved mortality outcomes as a result of increased hospital investment in counties hit by the 1968 flu.

A *social* channel would involve local behavioral shifts (e.g., in social distancing behaviors) in places that experienced high death rates from the 1968 flu, perhaps resulting in the development of a culture favoring extra precaution or social (dis)trust. In such cases, we would expect an equivalent shift for all people living within a geography (i.e., a population-level), as all residents would be more or less equally affected by these social forces.

An *individual* channel, in contrast, involves not a population but rather individuals for whom a difference in outcomes is expressed. The individual channel may be biological^2^ (e.g., learned immunity to the SARS-CoV-2 through prior exposure to another virus) or behavioral (e.g., individual-level compliance with public health measures), both of which may lessen the likelihood of infection or death. Along this line, Cheemarla et al. present evidence suggesting that individuals’ previous exposure to rhinovirus lends resistance to SARS-CoV-2 infection. The individual channel requires heterogeneous effects across a population, with sub-populations demonstrating unique COVID-19 outcomes based on past exposure.

### 2.2. Data

County-level mortality estimates of the 1968 influenza pandemic are derived from Centers for Disease Control and Prevention (CDC) Compressed Mortality files, 1968–1978, accessed via CDC’s WONDER database (43). We estimate the excess influenza death rate by comparing excess respiratory deaths in 1968 and 1969 (when the vast majority of 1968 flu deaths occur) to a baseline period of 1970 and 1971. We use death rates defined as deaths per thousand people using local population during a given time period. Our methodology of estimating excess mortality follows that used in previous work estimating mortality attributable to pandemic flu (44).

COVID-19 death rates come from a county-level dataset of COVID-19 deaths offered by the New York Times, based on reports from state and local health agencies (45). Included in counts are both confirmed and probable deaths, as categorized by states. The five county boroughs of New York City are grouped into one unit. We limit the analysis to the continental US.

Individual-level data are from CDC’s COVID-19 Case Surveillance Restricted Access Detailed Data accessed January 2021. The restricted dataset includes over 12 million records of COVID-19 cases with date, decadal age group, and county identifiers. Deaths are also reported. Note that because of CDC reporting delays and state-level data filing practices, aggregate totals are less than those of other sources and records November 2020 onward contain fewer counties reporting than earlier (46).

Hospital admission data are gathered from the US Department of Health and Housing Services (HHS). We aggregate weekly-level hospital data for different decadal age groups to the county-month level (47). Nursing home data come from the Centers for Medicare Medicaid Services (CMS) Nursing Home COVID-19 Public File. Nursing home facilities are required to self-report these data to the CDC.^3^ Patient-level healthcare data with year-of-birth information available from Healthjump via the COVID-19 Research Database consortium (50). The number of hospital beds at the county level are derived from the Centers for Medicare Medicaid Services. Mask use survey data come from the New York Times (51). County-level mobility data were made accessible to COVID-19 researchers by Google (52).

Covariate data include county-level race, ethnicity and age structure data from the US Census and mean county-level income data from the US Bureau of Economic Analysis (53, 54). Data on nursing home populations, incarcerated populations, uninsured populations, average household size, and work commuting methods come from the 2014–2018 American Community Survey (55, 56, 57, 58). Data on manufacturing establishments come from the American Economic Survey (59). Number of frontline workers were derived from CEPR data (60), transforming to county level assuming even allocation. The freight index is from the FHA’s Freight Analysis Framework (61).

### 2.3. Model

Our baseline linear regression model takes the following form:

(1) $${\text{outcome}}_{i}=\beta flu_{i}+\theta {\text{controls}}_{i}+\alpha_{s}+\epsilon_{i}$$

where *outcome*_*i*_ is a COVID-19 outcome in county *i* at a given month, *β* is the coefficient of interest related to *flu*, which is the excess respiratory death rate attributable to the 1968 flu as described in the Data section, *controls*_*i*_ is a vector of county-level covariates, *α*_*s*_ is a dummy for fixed effects in state *s*, and *ϵ*_*i*_ is the error term. Standard errors are clustered at the state level. For outcomes of interest we variously use (1) COVID-19 death rates, (2) hospital admissions, (3) a subset of patient-level data from Healthjump, and (4) nursing home death rates. All values are aggregated to the county level to match our data on 1968 flu intensity. We replicate this cross-sectional analysis at different snapshots in time representing the progression of the pandemic, as categorized by end-of-month outcomes.

To address endogeneity and omitted variable bias concerns in the relationship between the 1968 flu and COVID-19 outcomes, we also employ differences in death rates and hospital admissions by age group as the outcome variable. We choose the age cutoff based on whether someone was born before or after 1968 in an attempt to isolate those people likely to have been exposed to the 1968 pandemic.

The bottom panel of Figure 3 displays the identifying variation based on the age categorizations used in CDC’s COVID-19 case surveillance data and HHS’s hospital admissions data. This specification acts effectively as a difference-in-difference model to isolate the extent to which the 1968 flu affects people born before 1968 *relative* to those born after 1968. The identifying assumption relies on the fact that potential confounders are unlikely to shift the relative degree of COVID-19 morbidity or mortality across proximate age groups within a given county.

To address concerns about the coarse categorization of individuals into decadal birth cohorts, we validate the results with patient-level data from Healthjump containing annual year of birth.

## 3. Results

### 3.1. Aggregate COVID-19 mortality

Figure 1 shows cumulative COVID-19 death rates over time, averaged by age cohort and whether the county was among those severely hit by the 1968 flu pandemic. We see that death rates for the age groups 50–59 and 40–49 are similar, but the death rate among those aged 60–69 is consistently lower in high 1968-flu counties.

**Figure 2:**
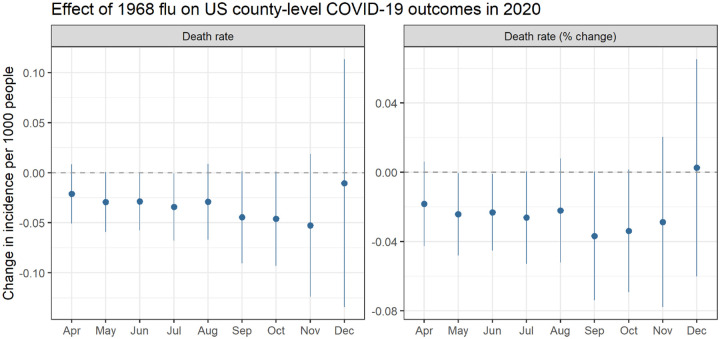
Relationship between county level excess death rates from the 1968 pandemic and COVID-19 death rates per 1,000 (left) and log-transformed death rates (right), which can be interpreted as percent change. For example, the September point estimate of −0.04 represents a 4% reduction. All models control for socioeconomic variables and state-level fixed effects. Coefficients are plotted from separately-run regressions for COVID-19 outcomes by month. Error bars reflect a 95% confidence interval.

**Figure A1:**
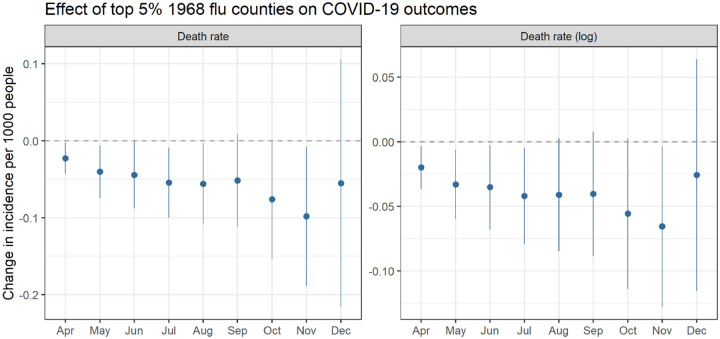
Relationship between counties in the top 5^th^ percentile of excess death rates from the 1968 pandemic and COVID-19 death rates (left) and log-transformed percent death rates (right) at the county level, controlling for socioeconomic variables and state-level fixed effects. Coefficients are plotted from separately-run regressions for COVID-19 outcomes by month. Error bars reflect a 95% confidence interval.

We next present regression results using the model specified in Equation 1. Figure 2 plots the coefficients representing the change in cumulative county-level COVID-19 death rates by month snapshot associated with an increase in 1968 flu mortality. We find a consistent negative relationship between the severity of 1968 outcomes and COVID-19 outcomes. Appendix Table A1 provides the full regression results including all the covariates, while Figure A1 utilizes a treatment dummy variable for counties greatly affected by the 1968 flu. In terms of magnitude, people in counties among the 10% worst hit by the 1968 flu had COVID-19 death rates 1–2% lower than the average US county.^4^

We test how health outcomes relate to known confounders, both in the present and in 1968. Appendix Table A2 presents the relationship of death rates from both COVID-19 and the 1968 flu on a selection of current and past covariates. Models (1) and (2) show results broadly in line with the COVID-19 literature: death rates are weakly correlated with income (negative), density (negative), the elderly population (positive), and Black population (positive). However, models (3) and (4) show that present-day risk characteristics are not correlated with outcome in 1968, which reduces concerns around confounding variables. Models (5) and (6) regress 1968 flu outcomes on historical covariates. We see that the size of the elderly population is a strong predictor of death rates, supporting past literature (36, 35). We also note a positive, but much weaker, relationship with a county’s Black population. This finding indicates that the 1968 flu had differential impacts by race in line with the disproportionate toll of COVID-19 on the Black community (62), as well as Hispanics and Native Americans (63).

**Figure A2:**
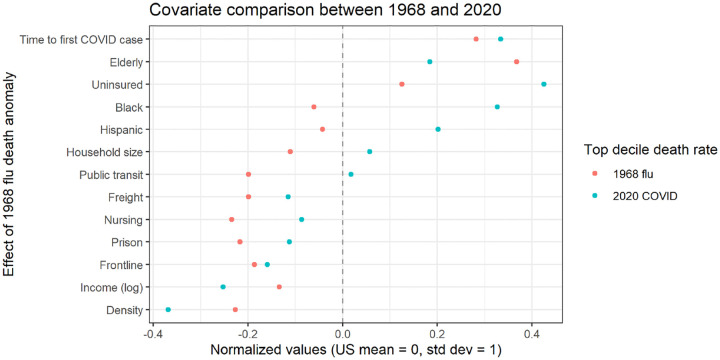
Plot of the mean values of the present-day covariates utilized in our baseline model. Means are separately computed for top decile counties in terms of both 1968 flu mortality and 2020 COVID-19 mortality. Values are normalized relative to a nation-wide mean of 0. We see a broad correlation in average county characteristics.

Appendix Figure A2 plots the mean values of the present-day covariates employed in our baseline model. Means are separately computed for top-decile counties in terms of both 1968 flu mortality and 2020 COVID-19 mortality. Values are normalized relative to a nationwide mean of 0. We see a broad correlation in average county characteristics. While not statistically different, there is gap in the average racial and ethnic composition such that COVID-19 had a more negative impact on Black and Hispanic communities relatively to the 1968 flu.

### 3.2. Death rates by age group

**Figure 3:**
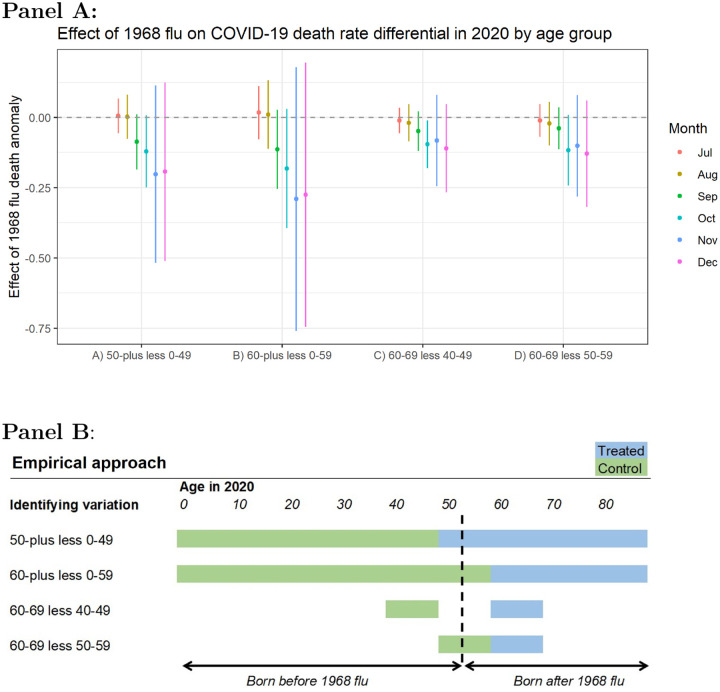
**Panel A:** Differences in county-level COVID-19 death rates per 1,000 across age groups associated with an increase in excess death rates from the 1968 pandemic, controlling for socioeconomic variables and state-level fixed effects. Coefficients are plotted from separately-run regressions for COVID-19 outcomes by month. Error bars reflect a 95% confidence interval. Panel B: Conceptual chart for the identifying variation used in the analysis. In the first row ‘50-plus less 0–49’ denotes the difference in the average COVID-19 death rate for the population age 50 or above (blue area) less the average death rate for the population under age 50 (green area). Multiple cutoffs are used given that the data is aggregated in decadal age brackets that do not align with exposure to the 1968 pandemic. The dotted line shows the theoretical age of someone in 2020 who was born in 1968.

Figure 3 plots the coefficients from Equation 1 using differences in COVID-19 death rates across age groups as the outcome variable based on CDC individual case data aggregated to the county level. There is a consistently negative point estimate for each of the four methods used to construct differentials around the age cutoffs. This means that COVID-19 death rates are lower among older cohorts relative to younger ones in places that experienced high 1968 flu mortality.

Columns (A) and (B) showcase the difference among all those older and all those younger than 50 and 60 years old, respectively. Columns (C) and (D) limit the span to single decades. For example, column (C) shows the difference in death rates between people in their sixties versus those in their forties, highlighting groups just old enough to have lived through the 1968 pandemic and those not. Column (D) uses people in their fifties as the control, although it is less obvious how to categorize this cohort, considering that many were alive in 1968 although flu transmission tends to increase after age five with the onset of schooling (64).

### 3.3. Hospital admissions

**Figure 4:**
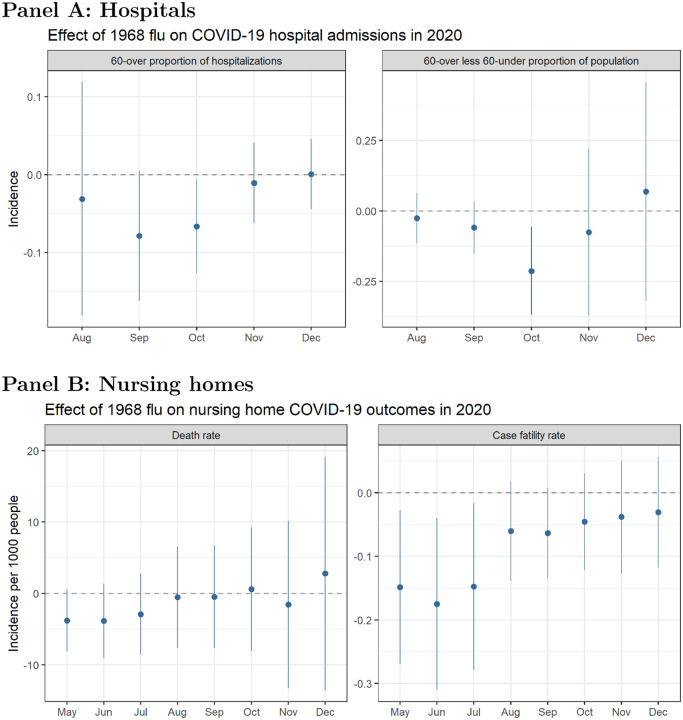
**Panel A:** Relationship between excess death rates from the 1968 pandemic and the proportion of COVID-19 hospital admissions among people over 60 (left) and the difference in the number hospitalized in the over-60 group relative to the under-60 group per 1,000 people (right). Rates are calculated based on cumulative COVID-19 hospital admissions at the county level. **Panel B:** Relationship between excess death rates from the 1968 pandemic and nursing home COVID-19 death rates, defined as cumulative deaths per 1,000 residents (left), and case fatality rate, defined as cumulative deaths as a proportion of cumulative COVID-19 cases (right). Models control for socioeconomic variables and state-level fixed effects. Coefficients are plotted from separately-run regressions for COVID-19 outcomes by month. Error bars reflect a 95% confidence interval.

In addition to examining death rates, we look at hospital admissions driven by COVID-19 as a proxy for case severity. We again test whether there is a differential effect of 1968 flu exposure on hospitalizations between people who lived through the pandemic and those who did not. The top panel of Figure 4 presents results in two ways: first, the proportion of COVID-19 hospital admissions among people over age 60, and second, the difference in the number hospitalized among those over 60 and those under 60 years old as a proportion of the population. The 1968 flu has a negative effect on hospital admissions for the over-60 group as a whole, as well as the difference in over-60 group relative to the under-60 group (i.e., the older group was hospitalized relatively less).

### 3.4. Nursing homes

We replicate our analyses using CMS data on nursing homes. The bottom panel of Figure 4 plots the effect excess death rates from the 1968 pandemic on COVID-19 death rates and case fatality rates (deaths as a proportion of cases) in nursing homes. There is a negative relationship between 1968 mortality and COVID-19 mortality, but the signal is much stronger for the case fatality rate.

### 3.5. Patient-level data

**Figure 5:**
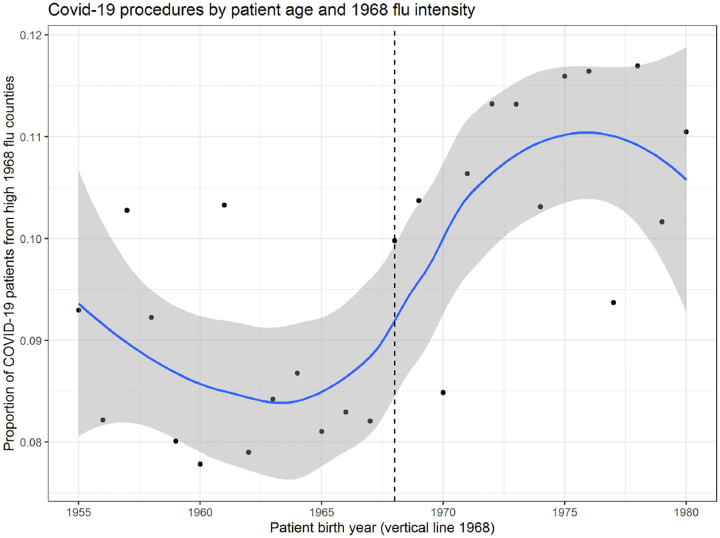
Plot of the ratio of patients in top decile 1968 flu counties relative to the count of all patients in the Healthjump dataset by patient year of birth. LOESS fit line shaded area representing a 95% confidence interval.

Because the prior two age group analyses involved decadal birth cohorts, we validate the results with patient-level data from Healthjump containing patients’ birth year. This analysis involves 48,000 unique patient records where a COVID-19 diagnosis is explicitly linked to a medical procedure within 30 days of the diagnosis. Such procedures include, but are not limited to, hospitalization. Figure 5 plots the ratio of patients in top 10% 1968 flu counties relative to the count of all patients. Older cohorts (people born before 1968) are better off in places hit hard by the 1968 flu, whereas younger cohorts are worse off. The discontinuity appears to be around 1968.

### 3.6. Behavioral and institutional responses

Next we test a number of potential relationships that would indicate a potential policy, social, or individual response to the 1968 pandemic.

#### 3.6.1. Hospital beds

One potential explanation is that places with high 1968 flu mortality subsequently invested in hospital capacity to be better prepared for future challenges. Such actions could explain why hard-hit counties saw better outcomes under COVID-19. To test this, Appendix Table A3 regresses present-day number of hospital beds at the county level on 1968 flu death rates and finds no relationship—in fact, the result is a precise zero under each model specification.

#### 3.6.2. Mask use

Another possibility is that individual behavior responds to an event like the 1968 flu. Adoption of risk-averse behaviors (whether on one’s own volition or induced through policy or social norms) could explain differential COVID-19 outcomes. Appendix Table A4 regresses a self-reported measure of mask use from the New York Times on county-level 1968 flu death rates and finds a negative relationship; that is, places worse hit by the 1968 flu tended to wear masks *less*. The relationship holds even after controlling for state-level differences and potential covariates (e.g., income, density, elderly, front line workers).

#### 3.6.3. Mobility

**Figure A3:**
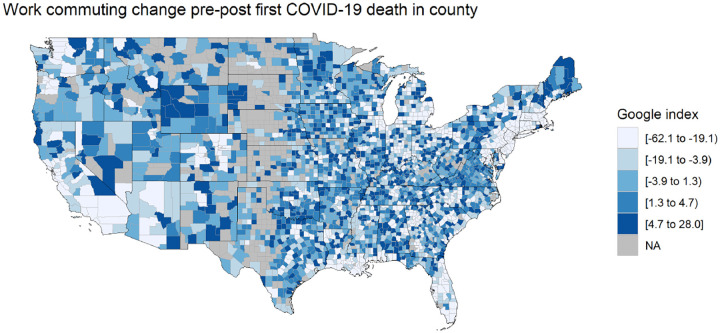
County-level map of Google mobility data on changes in time spent commuting to work 28 days before and after the first county death.

Finally, we report changes in mobility in response to 1968 flu death rates using Google mobility data. We look at both mobility involving time spent commuting to work as well as engaging in retail and recreational activities. We compute two measures to account for the potential timing of COVID-19 responses. One is a summer average of the baseline change in mobility in a county from June to August. Another is change in county mobility between 28 days before and 28 days after a counties’ first reported death. Appendix Table A5 shows a weak but positive relationship between 1968 flu deaths and mobility, suggesting that, if anything, behavior in counties adversely affected by the 1968 flu was less compliant with directives to minimize movement than other counties. Appendix Figure A3 shows a map of mobility changes in time spent commuting to work.

## 4. Discussion

Our findings suggest a persistent link between the 1968 flu pandemic and COVID-19 pandemic outcomes. People in counties among the 10% worst hit by the 1968 flu had COVID-19 death rates 1–2% lower than the average US county. This general relationship is robust to controlling for known confounders and holds across populations (i.e., county level aggregates, hospital admittees, and nursing home residents, subset of patients), as well as specifications that exploit age-based variation in exposure.

The direction of the relationship—locales with adverse outcomes in 1968 fare *better* today—mitigates concerns of a lurking omitted variable. In that case, we would imagine the opposite relationship in which some characteristic of places hit hard by the 1968 flu also makes them susceptible to COVID-19. Such a bias would indicate that the magnitude of our results are in fact understated.

It is worth noting that our results are generally strongest in the autumn of 2020. The effect of the 1968 flu on COVID-19 outcomes appears to fade into the winter as COVID-19 becomes widespread, a dynamic also seen in (20), and vaccines become available. Part of this may be attributable to the increasing number of US deaths that expands the number of counties making up the sample. In terms of the nursing home analysis, the more pronounced effect earlier in the COVID-19 pandemic may reflect an improvement in treatment of the disease over the course of the pandemic as the standard of care evolved and more therapeutics were incorporated into treatment (65).

We find evidence that the primary channel through which COVID-19 outcomes are affected is individual with a possibility of some unknown individual-level mechanism. To this end, we find no evidence of collective social activity suggesting better mitigation of COVID-19 outcomes. In fact, we find modestly lower levels of mask use and social distancing. Moreover, even if behaviors occurred that limited viral spread, the fact that positive outcomes were skewed toward age cohorts that lived through the 1968 flu pandemic suggests an individual rather than societal-level mechanism.

Focusing on nursing homes also allows us to isolate a population that—unlike the elderly living outside nursing homes—exercises less agency in their social distancing practices and other risk-mitigating behaviors, instead following nursing home policy. In addition, epidemiology within nursing homes is in some ways independent of what is found for the general population. Factors such as travel patterns of nursing home staff have a large effect on infection rates in such settings (66). We find that a smaller share of residents *who were infected* are dying. Assuming identical distributions of social distancing policy in nursing homes in counties with adverse 1968 flu histories and those without, better outcomes for nursing home residents in the former group are likely to suggest that some non-behavioral and non-policy mechanism is at play. This accumulated evidence lends support to a potential biologic mechanism driving differences in outcomes.

It is unclear what biologic mechanisms could be driving these findings, or whether some other unrecognized factors could be the force behind them. Direct immunological cross-reactivity seems unlikely, as SARS-CoV-2 coronavirus is a different type of virus than H3N2 influenza. However, individuals may have general innate, nonspecific, anti-viral immune pathways that are more robust due to factors such as genetics and lifestyle that may have been selected for during the 1968 pandemic and its aftermath. There is also precedent for generalized immune response experience early in life having an impact on susceptibility to future illness and injury, as has been observed in the aforementioned work examining the association between 1918 flu exposure and cardiovascular disease.

Thus some sort of selective pressure may have occurred over time in those places where people who survived the 1968 pandemic were generally better suited to survive the COVID-19 pandemic. This mechanism would not be unlike that identified in research linking tuberculosis outcomes to the 1918 flu pandemic and is plausible considering that 1968 flu mortality skewed toward younger people (26, 35). These mechanisms are purely speculative at this point, and significant research would be required to fully understand whether the differential outcomes observed here are truly meaningful and what caused them. Assessing the plausibility of cohort-based resilience to all respiratory diseases or whether this resilience is more specific to some element of COVID-19 that would otherwise cause death is certainly difficult, but may be worth considering as we try to further understand this pandemic in the coming years.

Our findings also motivate a discussion of broader social themes. It is noteworthy that the immediate institutional response to the 1968 pandemic was so muted. The US Surgeon General stated in 1969 that it was time to “…close the book on infectious diseases, declare the war on pestilence won” (34). Meanwhile, the 1968 pandemic lacks the salience of the 1918 pandemic, and recent retellings describe it as a “forgotten” pandemic (67).

Given its large health, economic, and societal impact, the COVID-19 pandemic will hopefully spur careful research into the costs and benefits of the dramatic public health measures taken worldwide, as well as potential structural and technological changes in society to mitigate or prevent future outbreaks of this magnitude. In light of our findings, further consideration should be given to the possibility that actions taken now could materially influence health outcomes during future pandemics—just as what happened in 1968 may have in some way reduced COVID-19 mortality among its survivors.

## Footnotes

1
https://www.cdc.gov/flu/pandemic-resources/1968-pandemic.html

2 An example of such a channel is how influenza affects age groups differently based on individuals’ past exposure to a similar viruses (36, 38, 39). Further, prenatal exposure to the 1918 influenza virus was linked to cardiovascular disease and other long-term effects (40, 41)

3 While the CDC successfully consolidated data from nursing homes across the US, there have been reports that the data received may not be comprehensive (48, 49)

4 Top decile counties had an average 1968 flu death rate of 0.54 per thousand compared to the US average of 0.07, a difference of 0.47. Multiplying this by the average of the coefficients on the right panel of Figure 2 (0.023) or the largest and most significant coefficient in September (0.037), yields a range of 0.011 to 0.017, or roughly 1.1–1.7%

## 5. Appendix

**Table A1:** Impact of 1968 flu on county COVID-19 deaths, cumulative by month

	Deaths per 1,000 as of month						
	Jun	Jul	Aug	Sep	Oct	Nov	Dec
	(1)	(2)	(3)	(4)	(5)	(6)	(7)
Flu deaths 1968	−0.029* (0.015)	−0.034* (0.017)	−0.029 (0.019)	−0.045* (0.023)	−0.046* (0.024)	−0.053 (0.036)	−0.010 (0.063)
Income per capita (log)	0.039 (0.031)	0.016 (0.030)	−0.053 (0.033)	−0.088** (0.042)	−0.152** (0.059)	−0.269*** (0.073)	−0.369*** (0.097)
Density	0.033 (0.022)	0.027 (0.024)	0.023 (0.026)	0.010 (0.026)	−0.009 (0.033)	−0.032 (0.041)	−0.122** (0.053)
Density-squared	−0.006 (0.005)	−0.005 (0.005)	−0.005 (0.005)	−0.004 (0.005)	−0.002 (0.006)	−0.001 (0.007)	0.007 (0.009)
Timing first case	−0.285*** (0.064)	−0.349*** (0.070)	−0.332*** (0.091)	−0.327*** (0.114)	−0.255** (0.115)	−0.152 (0.210)	−0.187 (0.250)
Elderly proportion	1.053*** (0.285)	1.302*** (0.270)	1.726*** (0.311)	2.015*** (0.397)	2.419*** (0.483)	2.970*** (0.640)	3.855*** (0.927)
Black proportion	0.846*** (0.172)	1.014*** (0.173)	1.176*** (0.170)	1.293*** (0.152)	1.289*** (0.147)	1.132*** (0.145)	0.865*** (0.181)
Hispanic proportion	0.139 (0.115)	0.347** (0.133)	0.660*** (0.204)	0.809*** (0.279)	0.884** (0.329)	0.971** (0.372)	1.075*** (0.385)
Freight intensity	0.007 (0.009)	0.009 (0.011)	0.009 (0.012)	0.010 (0.014)	0.011 (0.014)	0.015 (0.017)	0.023 (0.018)
Public transit proportion	0.016** (0.007)	0.015** (0.007)	0.014** (0.006)	0.012* (0.006)	0.014** (0.006)	0.017** (0.007)	0.015 (0.009)
Household size	0.138*** (0.051)	0.188*** (0.056)	0.199*** (0.063)	0.209*** (0.066)	0.189** (0.075)	0.077 (0.103)	−0.041 (0.141)
Nursing home proportion	0.00005*** (0.00001)	0.0001*** (0.00001)	0.0001*** (0.00001)	0.0001*** (0.00001)	0.0001*** (0.00001)	0.00005*** (0.00002)	0.00004** (0.00002)
Prisoner proportion	−0.00001* (0.00001)	−0.00001* (0.00001)	−0.00001* (0.00001)	−0.00001* (0.00001)	−0.00001 (0.00001)	−0.00001 (0.00001)	−0.00001 (0.00001)
Uninsured proportion	0.002 (0.002)	0.004 (0.003)	0.005* (0.003)	0.007** (0.003)	0.010** (0.005)	0.016*** (0.006)	0.021** (0.008)
Frontline proportion	−0.00000*** (0.00000)	−0.00000*** (0.00000)	−0.00000*** (0.00000)	−0.00000*** (0.00000)	−0.00000*** (0.00000)	−0.00000*** (0.00000)	−0.00000** (0.00000)
Controls	X	X	X	X	X	X	X
State FE	X	X	X	X	X	X	X
Observations	3,024	3,024	3,024	3,024	3,024	3,024	3,024
R^2^	0.416	0.437	0.478	0.464	0.382	0.335	0.331

*Notes:* Linear regression. Standard errors clustered at state level.

* p<0.1;

** p<0.05;

*** p<0.01

**Table A2:** Impact of current and past characteristics on COVID-19 and 1968 flu death rates

	*Dependent variable:*					
	COVID death rate		1968 flu death rate			
	–Current covariates–		–Current covariates–		–1969 covariates–	
	(1)	(2)	(3)	(4)	(5)	(6)
Income per capita (log)	−0.193 (0.140)	−0.372*** (0.095)	−0.024 (0.032)	0.002 (0.029)	0.022 (0.031)	0.044 (0.034)
Density	0.026 (0.106)	0.079 (0.055)	0.002 (0.009)	−0.002 (0.010)	0.001 (0.007)	0.001 (0.007)
Elderly proportion	1.333* (0.759)	1.549** (0.682)	−0.006 (0.182)	0.008 (0.192)	0.957*** (0.266)	1.099*** (0.245)
Black proportion	1.527*** (0.299)	1.070*** (0.207)	0.047 (0.047)	0.014 (0.052)	0.124** (0.050)	0.059 (0.067)
State FE		X		X		X
Observations	3,007	3,007	3,007	3,007	3,007	3,007
R^2^	0.109	0.298	0.001	0.030	0.012	0.039

*Notes:* Linear regression. COVID-19 deaths as of Nov 2020. We exclude covariates for which we lack data in 1968 to ensure similar sample composition. Standard errors clustered at state level.

* p<0.1;

** p<0.05;

*** p<0.01

**Table A3:** Impact of 1968 flu on current number of hospital beds

	*Dependent variable:*			
	Beds per 1,000			
	(1)	(2)	(3)	(4)
Flu deaths 1968	−0.088 (0.344)	−0.030 (0.326)	−0.009 (0.297)	−0.040 (0.314)
Controls			X	X
State FE		X		X
Observations	2,470	2,470	2,470	2,470
R^2^	0.00002	0.116	0.135	0.201
Adjusted R^2^	−0.0004	0.098	0.130	0.180
Residual Std. Error	4.322	4.104	4.032	3.913

*Notes:* Linear regression of county-level hospital beds per 1,000 people on excess 1968 flu death rates. Models control variously for socioeconomic variables and state-level fixed effects. Sample size reduced because not all counties have hospitals. Standard errors clustered at state level.

* p<0.1;

** p<0.05;

*** p<0.01

**Table A4:** Impact of 1968 flu on reported mask use frequency

	*Dependent variable:*			
	Mask use propensity			
	(1)	(2)	(3)	(4)
Flu deaths 1968	−0.017** (0.008)	−0.012** (0.005)	−0.013** (0.005)	−0.011** (0.005)
Controls			X	X
State FE		X		X
Observations	3,024	3,024	3,024	3,024
R^2^	0.002	0.374	0.248	0.419
Adjusted R^2^	0.002	0.364	0.244	0.407
Residual Std. Error	0.088	0.070	0.077	0.068

*Notes:* Linear regression of county-level mask use defined as proportion of sample responding to wearing masks ‘Sometimes’ or ‘Frequently’. Models control variously for socioeconomic variables and state-level fixed effects. Standard errors clustered at state level

* p<0.1;

** p<0.05;

*** p<0.01

**Table A5:** Impact of 1968 pandemic on mobility anomalies from Google

	*Dependent variable:*							
	Commuting				Retail			
	Summer average		Pre-post COVID death		Summer average		Pre-post COVID death	
	(1)	(2)	(3)	(4)	(5)	(6)	(7)	(8)
Flu death 1968	1.100** (0.540)	0.774 (0.550)	2.983*** (1.092)	2.870*** (0.965)	6.640** (3.100)	4.269 (2.666)	5.301* (2.820)	4.114 (2.566)
Covariates		X		X		X		X
State FE	X	X	X	X	X	X	X	X
SE Cluster	State	State	State	State	State	State	State	State
Observations	2,719	2,719	2,651	2,651	1,757	1,757	1,697	1,697
R^2^	0.162	0.474	0.185	0.444	0.195	0.403	0.174	0.376
Adjusted R^2^	0.147	0.461	0.170	0.431	0.173	0.381	0.150	0.352
Residual Std. Error	6.154	4.890	13.184	10.917	14.877	12.867	19.147	16.717

*Notes:* Linear regression of average mobility responses from data provided by Google. Models (1)–(4) involve mobility in relation to commuting to work, and models (5)–(8) involve retail and recreational activities. In models (1), (2), (5), and (6), Summer average is the average baseline change in mobility in a county over June to August. In models (3), (4), (7), and (8), Pre-post COVID death is the change in county mobility between the periods 28 days before and 28 days after a counties’ first reported death. Models control variously for socioeconomic variables and state-level fixed effects. Observations missing for counties and months with not enough activity for Google to provide an estimate. Standard errors clustered at state level.

* p<0.1;

** p<0.05;

*** p<0.01
